# Comparison of four techniques on facility of two-hand Bag-valve-mask (BVM) ventilation: E-C, Thenar Eminence, Thenar Eminence (Dominant hand)-E-C (non-dominant hand) and Thenar Eminence (non-dominant hand) – E-C (dominant hand)

**DOI:** 10.15171/jcvtr.2016.30

**Published:** 2016-12-27

**Authors:** Maryam Soleimanpour, Farzad Rahmani, Alireza Ala, Hamid Reza Morteza Bagi, Ata Mahmoodpoor, Samad EJ Golzari, Fatemeh Zahmatyar, Robab Mehdizadeh Esfanjani, Hassan Soleimanpour

**Affiliations:** ^1^Social Determinants of Health Research Center, Tabriz University of Medical Sciences, Tabriz, Iran; ^2^Emergency Medicine Research Team, Tabriz University of Medical Sciences, Tabriz, Iran; ^3^Anesthesiology Research Team, Tabriz University of Medical Sciences, Tabriz, Iran; ^4^Medical Philosophy and History Research Center, Tabriz University of Medical Sciences, Tabriz, Iran; ^5^Students’ Research Committee, Tabriz University of Medical Sciences, Tabriz, Iran; ^6^Neurosciences Research Center, Tabriz University of Medical Sciences, Tabriz, Iran; ^7^Road Traffic Injury Research Center, Tabriz University of Medical Sciences, Tabriz, Iran

**Keywords:** Bag-Valve-Mask Ventilation, Airway Management, Two Hand BVM Ventilation

## Abstract

***Introduction:*** Bag-valve-mask (BVM) ventilation is the first and important part of the airway management. The aim of present study was to evaluate the quality of four different BVM ventilation techniques – E-C, Thenar Eminence, Thenar Eminence (Dominant hand)-E-C (Non dominant hand), and Thenar Eminence (Non dominant hand)-E-C (Dominant hand) – among two novice and experienced groups.

***Methods:*** In a case-control and mannequin based study that was conducted in Tabriz University of medical sciences, 120 volunteers were recruited and divided into two groups. 60 participants in experienced and other 60 as novice group who observed BVM ventilation but hadn’t practical experience about BVM ventilation. Every participant in both groups performed 4 BVM ventilation techniques under the supervision of an experienced assessor. Quality of mannequin chest expansion was recorded by two other experienced assessors who were blind to ventilation process. The data were analyzed with SPSS 17.0.

***Results:*** In novice group, when evaluating each technique performance, they did Thenar Eminence (non-dominant hand) - E-C (dominant hand) technique much better than the others (*P*<0.0001). But in the experienced group, there was no meaningful difference between the all four techniques (*P*= 0.102).

***Conclusion:*** Novice participants did Thenar Eminence (non-dominant hand) - E-C (dominant hand) technique better than the others. Therefore, it is recommended that training of this technique was placed in educational program of medical students.

## Introduction

**Figure 1:**
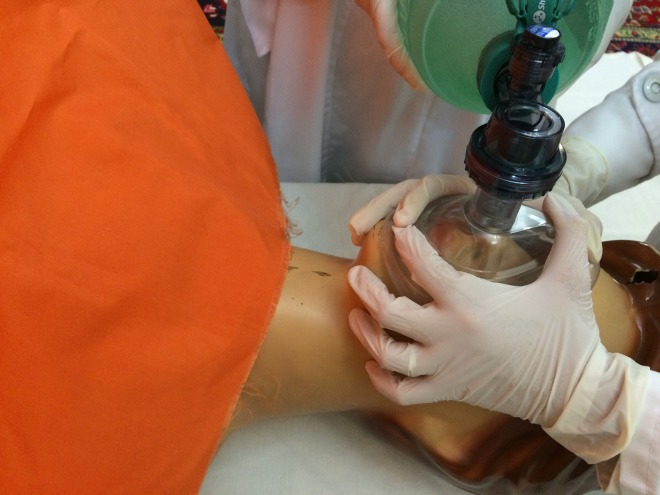
Two hand Bag-mask ventilation techniques (E-C technique).

**Figure 2:**
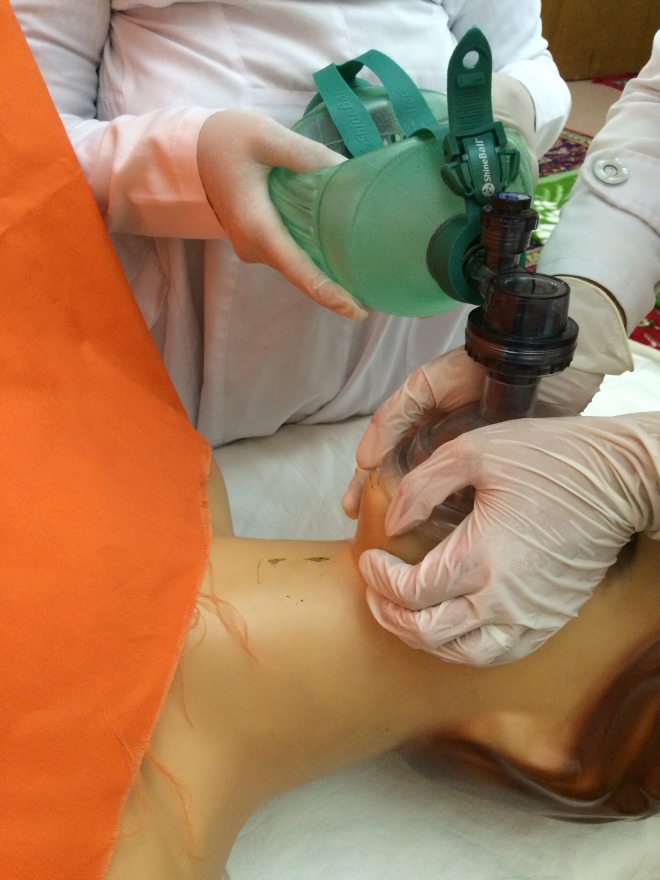
Two hand bag-mask-ventilation techniques (Thenar eminence technique).

**Figure 3:**
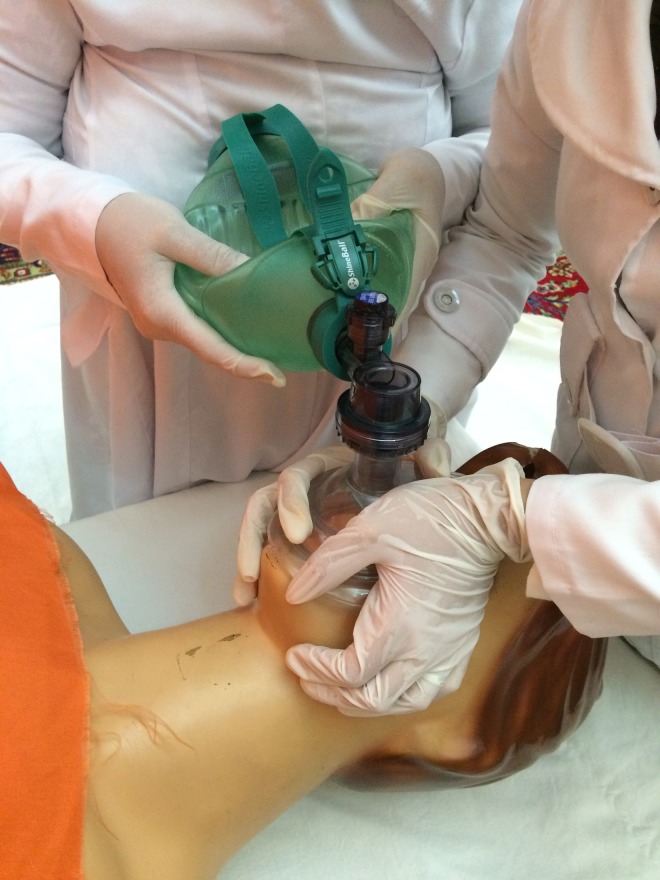
Two hand bag-mask-ventilation techniques (E-C and thenar eminence technique).

Bag-valve-mask (BVM) ventilation is one of the major principals of maintaining airway in different medical scenarios.^1-4^ Although, BVM ventilation seems to be easy to perform, its proper application could be challenging, especially with less experienced medical staff. In the most broadly used technique, E-C clamp technique, the applicant holds and fixed the mask on the face using one hand and ventilation is achieved through pressing the bag with the other hand (Figure 1). Usually, in order to reach the required ventilation, the applicant performs simultaneous jaw thrust using the third, fourth and fifth fingers (resembling E letter) while vertical pressure is applied on the mask using thumb and index fingers (resembling C letter). Despite being extremely advantageous in resource-limited settings, the staff using this method needs to be experienced unless the required mask fitting on the face would not be achieved and consequently, the ventilation would not be secured. Alternatively, Thenar Eminence technique could be used in which is applied bimanually and therefore another person is required to push the bag (Figure 2).^4-10^ Having more than 55 years old age, male gender, body mass index (BMI)> 31 kg/m^2^, anesthesia provider, obstructive sleep apnea (OSA), moustache, a short neck, history of neck radiation, short thyromental distance, Mallampati score of 3 and 4 are mentioned as risk factors of difficult BVM ventilation.^7^ Golzari et al showed that placing folded sterile gas in buccal cavities in toothless patient scan remarkably improve BVM ventilation when compared to those toothless individuals with or without denture.^2^ In the present study, we compared the efficacy of two new combined techniques with these two conventional methods in two groups of the experienced and novice staff on mannequin. In new combined techniques, the participant used E-C technique with one hand and simultaneously Thenar Eminence technique with the other hand (Figure 3). Later, the participant switched the techniques and hands. Through analysis of obtained results, it was tried to choose the simplest way of BVM ventilation for novice practitioners.

## Materials and Methods

The study was in a type of case-control one that was carried out on the mannequin in the skill lab of Tabriz University of Medical Sciences. To calculate the sample size, we had a pilot study on 11 individuals for each group. Each member of both groups did 4 E-C, Thenar eminence, Thenar eminence (dominant hand) - E-C (non-dominant hand), Thenar eminence (non-dominant hand) - E-C (dominant hand) techniques. Two assessors scored them from 1 to 4.

Score 1: no appreciable chest expansion

Score 2: minimal chest expansion

Score 3: moderate chest expansion

Score 4: good chest expansion

Scores 3 and 4 were acceptable while 1 and 2 were unacceptable. After calculation of scores, following results gained: Group 1 (experienced) performed all 4 techniques in a complete way. But group 2 (novice) did E-C 63% acceptable (7 out of 11), Thenar eminence with 82% acceptability (9 out of 11), Thenar eminence (dominant hand)- E-C (non-dominant hand) with 73% (8 out 0f 11) and Thenar eminence (non-dominant hand)–E-C (dominant hand) with 82% (9 out of 11). Considering 90% power, the sample size was calculated according to sample size formula with compare the mean of both groups.The highest sample size was calculated to be 48 cases, we increased the number to 60 cases for increasing validity of the study.

[n=(Z_α/2_+Z_β_)^2^ * (P_1_(1-P_1_)+ P_2_(1-P_2_)) / (P_1_-P_2_)^2^]

In which

P_1_=1, P_2_=0.82, Z_α/2_=1/96, Z_β_=1.28

After the approval by Ethic Committee of the university, we undertook our study on participants. First of all, participants were screened. Then consent was obtained and they were explained about the safety of the program. Inclusion criteria were academic board staff, residents, and interns of anesthesiology and emergency medicine departments. Exclusion criterion was also their dissatisfaction to enter the study. Group 1 included 60 participants who could perform BVM ventilation easily. They were anesthesiologist and emergency medicine scientific board and senior residents who had more than 3 years job experience and master in BVM ventilation. Group 2 included 60 emergency medicine and anesthesiology interns who observed BVM ventilation but did not have practical experience. At first, mannequin was divided from neck and chest with a blurred curtain. In a way that a person who perform BVM ventilation could not see mannequin chest. The assessors stood on the other side of curtain and they could not see the ventilation process. Each attending observed the other departments’ interns and residents then the anesthesia or emergency medicine attending did not know the person who is performing the ventilation. Every participant followed 4 techniques with two hands according to computer randomized selection. In brief, each participant did all 4 BVM ventilation techniques while an experienced assessor who was emergency medicine attending stood on his dominant hand side and told him to follow sniffing position maneuver during ventilation process. Moreover, the attending was responsible for ventilating and deflation by both groups and did deflation process in a second (two times ventilation and each per second). Two other assessors who were experienced emergency medicine or anesthesiology attending were in front of participant and who was blinded to ventilation process but observed mannequin chest expansion during ventilation (two times ventilation and each per second). The efficacy of the ventilation was evaluated by the two attending professors who were blinded to participant observing the two times ventilation and chest expansion (1 second/breath) using a four scale scoring system as followings^6^:

Score 1: No appreciable chest expansion

Score 2: Minimal chest expansion

Score 3: Moderate chest expansion

Score 4: Good chest expansion.

Ventilation efficacy was considered as ideal, average and weak if the achieved scores were (4-4), (3-3 or 3-4 or 4-3) and (1 or 2 at any of the attempts), respectively.^4^ Then, the results were analyzed through SPSS 17.0 software and they were compared with other techniques with chi-square test. Level of meaningfulness also considered to be *P *< 0.05.

## Results

Experienced participants performed both conventional techniques significantly better than the novice participants (Table 1). However, no significant difference could be observed between both groups using the two novel combined methods (Table 1). Interestingly, statistically significant differences could be observed in novice group regarding the four different BVM ventilation techniques. The participants in novice group performed the Thenar Eminence (non-dominant hand)/E-C (dominant hand) the best (*P* < 0.0001). Nevertheless, no significant difference could be observed among the participants in experienced group.

**Table 1:** Comparison of four techniques

**Technique**	**Volunteer**	**Ideal**	**Average**	**Weak**	***P*** ** value**
E–C	Experienced	13 (21.7%)	35 (58.3%)	12 (20%)	0.003
	Novice	3 (5%)	31 (51.7%)	26 (43.3%)	
Thenar Eminence	Experienced	23 (38.3%)	28 (46.7%)	9 (15%)	0.007
	Novice	8 (13.3%)	38 (63.3%)	14 (23.3%)	
Thenar Eminence (dominant hand) E-C (Non dominant hand)	Experienced	9 (15%)	39 (65%)	12 (20%)	0.618
	Novice	13 (21.7%)	37 (61.7%)	10 (16.7%)	
Thenar Eminence (Non dominant hand) E-C (Dominant hand)	Experienced	17 (28.3%)	36 (60%)	7 (11.7%)	0.395
	Novice	20 (33.3%)	37 (61.7%)	3 (5%)	

## Discussion

According to American Heart Association (AHA), learning cardiopulmonary resuscitation (CPR) is an urgent and fundamental priority to everyone in the world.^11^ It seems more vital when we talk about medical working staffs. BVM ventilation is one main part of CPR. Even, sometimes, it can be more vital than intubation, especially when there is a disorder or severe trauma in airways or lack of experience in intubation procedure (that are called difficult airway).^9,12^ When faced with this situation, performing appropriate ventilation with BVM would be very helpful (although, nowadays, laryngeal mask airway [LMA] has been considered as an alternative to manage difficult airways).^10^ Thus we decided to undertake and study to improve ventilation process in addition to skilled persons, novice people would also successfully perform ventilation. Therefore we concluded that novice participants can skillfully do two hand ventilation with BVM in which E-C is dominant and Thenar Eminence is non dominant. Airway management was one high priority for physicians of all ages.^13^ Every doctor must be proficient of managing airways.^14^ Novice individuals (general practitioners) can be taught promptly and be a potential source to save people’s life by handy techniques such as BVM ventilation in critical condition.^15^ Various investigations have been done about how to ventilate with BVM (we should take into the consideration the very important point that patient always dies from lack of oxygen, not because of the inability to be intubated). In a study by Racine et al upon 46 toothless patients it was concluded that by tightening mask on lower lip with two hands, the air leak can be prevented significantly compared to the common positioning of the mask in which mask padding is placed on the chain.^5^ In another study by Taxak et al, they showed that using a long rolled up gauze that covers both vestibules of the oral cavity in a packed way can improve BVM ventilation in toothless patients.^16^ In another investigation by Conlon et al on 166 toothless patients it was confirmed that BVM ventilation is improved in patients with denture,^17^ it was also depicted in Golzari et al study that putting rolled up gauze in vestibules of oral cavity of toothless patients can significantly improve BVM ventilation compared to edentulous patients with denture.^2^ Takashi et al used dental adhesives in patients with the nasogastric tube to improve BVM ventilation. Results showed that application of dental adhesives in those patients can increase expiration volume compared to other times.^2^ In addition to BVM ventilation techniques there are other factors can cause to difficult BVM ventilation and Langeron et al identified this problematic factors i.e., age >55 years, BMI > 26 kg/m^2^, beard, edentulous patients, history of snoring.^18^ Adent tried to compare and collect presented articles of the field and suggested that a scoring system about BVM ventilation must be established.^19^ Finally, Han et al formulated a scoring system from 1 to 4 in which scores 3 and 4 were respectively belonged to difficult and impossible BVM ventilation.^20^ Umesh et al in his research compared E-C with E-O in which first and second finger like O letter covers mask entrance hole and three other fingers cover chain. They concluded that novice people can do E-O BVM ventilation rather better than E-C technique.^6^ According to a report by American Society of Anesthesiology (ASA), BVM ventilation problem occurs when anesthesiologist cannot manage ventilation due to one or more of the issues such as insufficient coverage of mask on face, air leak or high resistance to air stream.^4^ Therefore, a critical point to have efficient BVM ventilation is appropriate coverage of mouth and nose with the mask. There are 3 main problems with adequate ventilation. These problems are inadequate air stream, lack of oxygen and gastric distention. So appropriate cover of nose and mouth with the mask can eliminate two first problems and can be minimized by two hand BVM ventilation.^3^ According to the instructions by AHA two hands BVM ventilation is prior to one hand type.^11^ Therefore, in the present research, we adopted a novel two hand BVM ventilation technique on both novice and experienced groups on the mannequin. The advantage of our study over the studies like Umesh et al^6^ is that in their study ventilation was performed with one hand. In present work it is actually tried to study ventilation process with a novel two hand BVM ventilation technique in which the a person who perform BVM ventilation has his dominant hand in E-C mode and non-dominant hand in Thenar eminence and then vice versa and then we compared it with E-C technique and Thenar Eminence among two novice and experienced groups. By doing this we tried to introduce the easiest technique for novice people. Assessment of ventilation quality in the present study was completely objective. It was not, however, possible to implement paraclinical techniques like tidal volume assessment, or maximum airway pressure to assess ventilation quality. These were our study limitations.

## Conclusion

We finally concluded that experienced group performed all 4 techniques at the same level of proficiency and no statistical significant difference was observed between the techniques. All obtained results from experienced group were acceptable. On the contrary, novice group did Thenar eminence (non-dominant hand)-E-C (dominant hand) much better than the others. As Thenar Eminence (Non-dominant hand)/E-C (dominant hand) technique was performed with the highest efficacy in novice participants, this novel combined method is suggested to be used throughout the education of the less experienced medical staff.

## Competing interests

The author(s) declare that they have no competing interest.

## Ethical Approval

The institutional ethics committee approved the study protocol.

## Acknowledgments

The authors are grateful to all participated in the study, in addition to data collectors, supervisors, and administrative staff of Skill Lab of Medical Faculty, Tabriz University of Medical Sciences, Tabriz, Iran. This article was written based on dataset of Fatemeh Zahmatyar’s Medical thesis entitled” Comparison of Four techniques on facility of Two-Hand Bag-Mask-Ventilation: E-C,Thenar Eminence, Thenar Eminence (Dominent hand)-E-C(Non-Dominant hand) and Thenar Eminence (Nond-Dominent hand) – E-C (Dominant hand),” registered in Tabriz University of Medical Sciences (No. 25- June 28, 2014) and was presented in October 2015. This article was supported by Road Traffic Injury Research Center, Tabriz University of Medical Sciences, Tabriz, Iran. Special thanks to Research Vice Chancellor of Tabriz University of Medical Sciences for all the material and financial support in our study.
